# THE FEASIBILITY, ACCEPTABILITY, AND APPROPRIATENESS OF A PERSON-CENTERED COMMUNICATION TOOL

**DOI:** 10.1093/geroni/igz038.195

**Published:** 2019-11-08

**Authors:** Shamatree Shakya, Heshuo Yu, Emily Mueller, Kimberly Van Haitsma, Katherine Abbott

**Affiliations:** 1 Miami University, Oxford, Ohio, United States; 2 Scripps Gerontology Center at Miami University, Oxford, Ohio, United States; 3 The Pennsylvania State University, University Park, Pennsylvania, United States; 4 Miami University: Scripps Gerontology Center, Oxford, Ohio, United States

## Abstract

The purpose of this study was to assess the acceptability, feasibility, and appropriateness of the PAL Card intervention. The data for this study came from monthly logs and final calls completed by n=26 NH providers. Staff from multiple departments contributed to the PAL card implementation. Common places for placing PAL cards were wheelchairs, walkers, doors, and in closets. Over 90 % of residents approved of the accuracy of information presented in PAL cards. From the providers’ perspective, PAL cards’ acceptability ranged from 96 to 100%, appropriateness ranged from 93.10 to 100 % and feasibility ranged from 90 to 100%. The total staff time estimated costs for PAL card implementation ranged from $180 to $3,236 depending on the individuals involved. The PAL card intervention was deemed acceptable, feasible, and appropriate by providers and accurate by residents. A discussion of the opportunity costs associated with implementing this intervention will be discussed.

